# The influence of gravity on respiratory kinematics during phonation measured by dynamic magnetic resonance imaging

**DOI:** 10.1038/s41598-021-02152-y

**Published:** 2021-11-25

**Authors:** Louisa Traser, Carmen Schwab, Fabian Burk, Ali Caglar Özen, Michael Burdumy, Michael Bock, Bernhard Richter, Matthias Echternach

**Affiliations:** 1 Institute of Musicians’ Medicine, Faculty of Medicine, Medical Center – University of Freiburg, Freiburg, Germany; 2 Department of Prosthetic Dentistry, Center for Dental Medicine, Faculty of Medicine, Medical Center – University of Freiburg, Freiburg, Germany; 3 Department of Otorhinolaryngology, Head and Neck Surgery, University Medical Center Hamburg-Eppendorf, Hamburg, Germany; 4 Department of Radiology, Medical Physics, Faculty of Medicine, Medical Center – University of Freiburg, Freiburg, Germany; 5 Division of Phoniatrics and Pediatric Audiology, Department of Otorhinolaryngology, Munich University Hospital, Munich, Germany

**Keywords:** Physiology, Respiration

## Abstract

Respiratory kinematics are important for the regulation of voice production. Dynamic MRI is an excellent tool to study respiratory motion providing high-resolution cross-sectional images. Unfortunately, in clinical MRI systems images can only be acquired in a horizontal subject position, which does not take into account gravitational effects on the respiratory apparatus. To study the effect of body posture on respiratory kinematics during phonation, 8 singers were examined both in an open-configuration MRI with a rotatable gantry and a conventional horizontal MRI system. During dynamic MRI the subjects sang sustained tones at different pitches in both supine and upright body positions. Sagittal images of the respiratory system were obtained at 1–3 images per second, from which 6 anatomically defined distances were extracted to characterize its movements in the anterior, medium and posterior section of the diaphragm as well as the rip cage (diameter at the height of the 3rd and 5th rip) and the anterior–posterior position of the diaphragm cupola. Regardless of body position, singers maintained their general principles of respiratory kinematics with combined diaphragm and thorax muscle activation for breath support. This was achieved by expanding their chest an additional 20% during inspiration when singing in the supine position but not for sole breathing. The diaphragm was cranially displaced in supine position for both singing and breathing and its motion range increased. These results facilitate a more realistic extrapolation of research data obtained in a supine position.

## Introduction

The breathing apparatus is one of three functional units that regulate voice production together with vocal fold oscillations and the vocal tract (VT). Its primary task is the regulation of subglottic pressure, caused by the expiratory force of the respiratory system applied to the vocal folds. It determines the fundamental frequency and loudness of phonation and must be tailored individually for each note to be sung in tune at an intended loudness^1^. This is mainly achieved by activation of different breathing muscles with the goal to supplement or overcome the elastic recoil forces^2^. The activation of the breathing muscles in turn has a direct impact via the tracheal pull on the vertical laryngeal position and thus vocal fold oscillation and vocal tract resonance^3^. Thus, a dysfunction of the respiratory system during voice production can be related to voice disorders^4–7^. To improve voice quality in voice therapy or singing pedagogy the breathing apparatus is often a promising therapeutic target. However, the underling principles of economic and efficient function of the respiratory system during voice production are still not understood in detail.

Technical advances in imaging technology provide new insights into the functional processes of the body. Magnetic resonance imaging (MRI) in particular has been shown to be advantageous as it offers 2D and 3D imaging capabilities in all spatial directions with an excellent contrast between soft tissues, and it enables dynamic imaging without harmful ionizing radiation, which makes it an ideal imaging modality for studies in healthy subjects. In two pilot studies, the respiratory system of professional singers was dynamically imaged using MRI during sustained phonation^8^ and pitch jump phonation^9^. During singing, a differentiated pattern of movements of the respiratory system was revealed, which was very different from pure exhalation: for continuous adjustment of subglottic pressure to the prevailing passive recoil forces, a combined adaptation of inspiratory activity occurred in the thorax and diaphragm. At the beginning of sustained phonation the posterior and medial section of the diaphragm (DPH) elevated quickly, while the anterior section and thorax exhibited a slower movement, with the opposite pattern measured at the end of phonation^8^. As both the DPH and the thorax are included as regulatory compartments in the adaptation of subglottic pressure, this concept is also called “mixed” inspiratory slowing of the respiratory movement. Contractions of the posterior DPH were visualized for phonation of downwards pitch jumps^9^.

However, MRI studies have the distinct disadvantage that measurements are typically taken in a supine body position with a horizontal patient table. Studies on lung function reported posture related differences with an increase of functional residual capacity in the supine body position^10,11^ while vital capacity (VC) and forced VC decreased^12^. The range of motion of the DPH, on the other hand, increased during respiration in the supine position^13^. This seems justifiable, since gravity acts on the DPH and abdomen in the inspiratory direction in the upright position, since it enlarges the abdominal cavity^14^. At the same time, it reduces the size of the thorax, which causes it to act in the expiratory direction^7,14^. In a supine position, however, gravity acts in the expiratory direction on both compartments. The DPH can compensate to some extent for the associated cranial displacement of the abdominal contents by increasing its contractile force. This in turn fits that Hixon et al. described a fundamental change in the respiratory kinematics for phonation in a supine body position from mixed to sole DPH mediated inspiratory activation in untrained subjects^15^. A change in the respiratory strategy for breath support during phonation, as described by Hixon et al. would strongly question the transferability of results obtained in the supine position to the upright position. However, as gravity was found to be less influential on professional singers’ vocal tract configuration during singing^16,17^, it is possible that trained singers and non-singers also differ in their sensitivity to posture-related effects for breath support.

Characterization and quantification of a posture related effect on respiratory kinematics in singers has not yet been performed because comparative imaging in the upright body position is possible only in a few specialized scanners that are not widely available. As a consequence of the open magnet design of these scanners the field of view is reduced and low gradient performance of these devices additionally lead to a limited temporal and spatial resolution. Still, technical advances now allow the dynamic imaging of the respiratory system during singing phonation in an upright position. The presented study therefore aims to visualize, characterize and quantify position-dependent changes in respiratory kinematics of professional singers for the first time. The aim of this study is to enable a more realistic extrapolation of research results obtained of a supine position, as well as to consider the implications of these findings for voice pedagogy.

## Methods

### Subjects

This study was approved by the Medical Ethics Committee of the University of Freiburg, (Nr.273/14). All participants gave informed consent prior to the investigation. This study was performed in accordance with all relevant guidelines and regulations.

Professional singers were chosen as subjects because they have very consistent and economic breathing strategies^18,19^, and they can be regarded as a good model for effective voice production. Furthermore, through education and training, they are assumed to be less distracted by the noise during the MR imaging as they are used to auditory masking (e.g., in choir singing). Professional singers are also used to singing in different body positions in director’s theatre. Their employment of very consistent breathing motions^18,19^ is especially important for this study because respiratory movements are being compared in different body positions and MRI systems.

Eight singers who were trained in western classical singing were enrolled in this study. Six of the eight subjects (Nr. 1–5,8) were professional singers with singing as their sole income, while two subjects were experienced semi-professional singers (Nr. 6&7). Subjects 1–3 were also part of a pilot study^8^. Supplementary Table S1 online shows the subjects’ age, gender, voice classification, classification according to the Bunch and Chapman taxonomy, i.e., a classification of professionalism in singers^20^, and relevant physical characteristics (vital capacity (VC), forced expiratory volume in one second (FEV1), height, weight). VC and FEV1 were obtained in a clinical setup using a ZAN100 spirometer (ZAN, Oberthulba, Germany) according to^21^ in upright and additionally supine body position. At the time of the recording and MRI measurements, none of the participants had any vocal complaints, history of voice disorders, or respiratory pathologies.

### Tasks

The phonation tasks were chosen according to the pilot study^8^ and voice classification of the singer. Subjects were asked to sustain each pitch on vowel [a:] for as long as possible (maximum phonation time, MPT) at a medium loudness (mezzo forte, mf). This was repeated at three different fundamental frequencies which represent a low (P1), medium (P2) and high pitch (P3) in the tessitura of the respective repertoire of the singer. The singers were additionally asked to phonate P2 in two in additional loudness conditions (soft phonation = pianissimo *pp*, and loud phonation = fortissimo *ff*). Additionally, the singers were asked to breathe in and out to the greatest extent to assess their vital capacity. The protocol is additionally given in Supplementary Table S2 online. The pitch was presented via headphones directly before the task, and the subjects could repeat the task until they were satisfied with the outcome. For evaluation only the best version was used.

### Magnetic resonance imaging

The imaging of the singers’ breathing apparatus was performed using two different MRI systems, that offer MRI in either a supine or an upright position:

#### Conventional horizontal MRI (= hMRI)

The first system was a clinical whole-body 1.5 T MRI system (Tim Symphony, Siemens, Erlangen, Germany) which was also used in a pilot study^8^. The subject positioning and measurement were here only possible in supine body positioning (referred to as supine *hMRI*). In accordance with the pilot study, a 3D localizer data was first applied to position a sagittal slice in the right lung in which the vertex of the DPH cupola and the apex of the lung could be identified. Then, a dynamic 2D trueFISP imaging sequence (repetition time/ echo time (TR/TE) = 3/1.5 ms, *α* = 6°, bandwidth (BW) = 977 Hz/px, slice thickness (ST) = 10 mm, acquisition matrix = 256, field of view (FOV) = 420 mm) was applied with a temporal resolution of approximately 3 frames per second (fps) while the singer performed each singing task. The subjects wore headphones for hearing protection and communication during the session.

#### Upright and Supine rotatable MRI (= rMRI)

**Figure 1:**
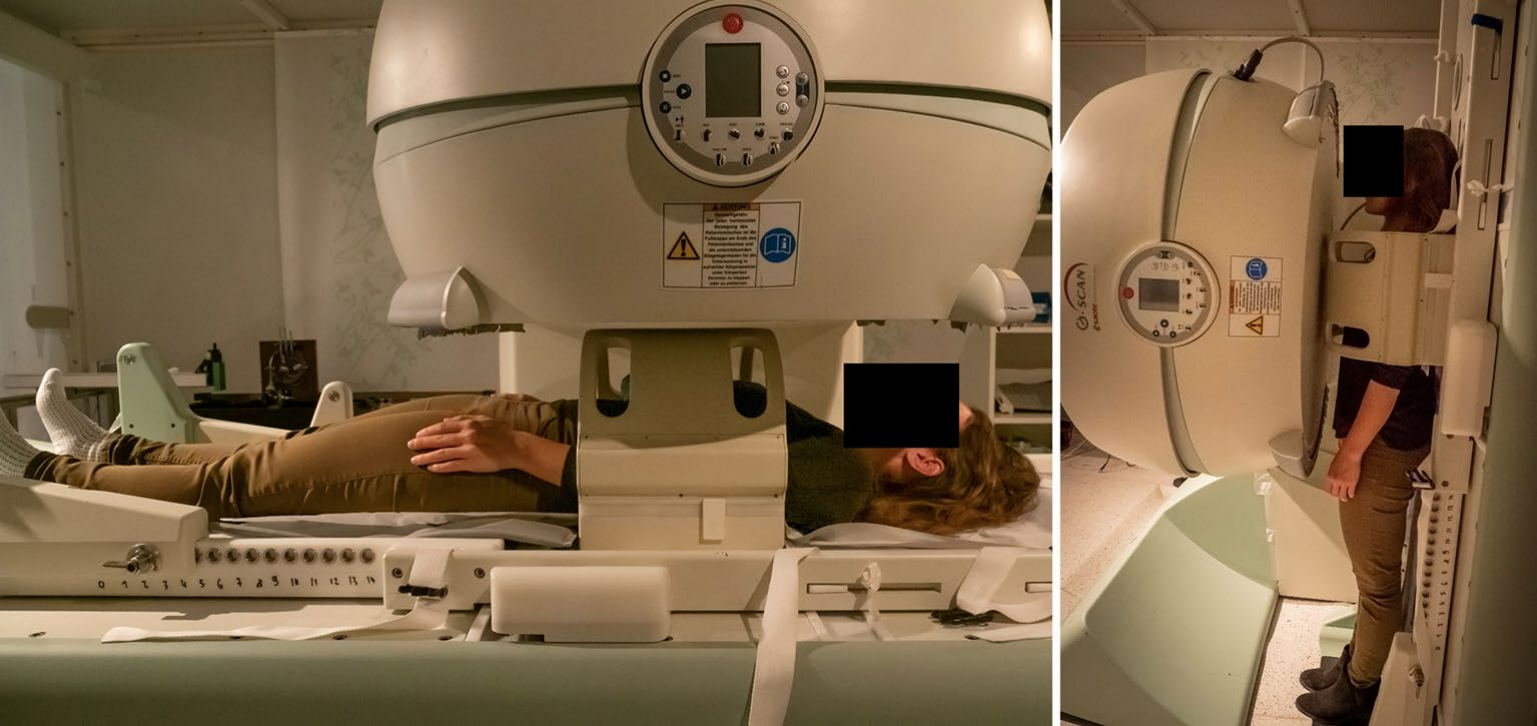
Subject positioning in the rotatable MRI system. Subjects were either placed in a lying supine (0°, left) or in an upright standing (80°, right) position.

Directly after the supine MRI a second upright measurement was performed in an open-configuration, weight-bearing MRI system with a rotatable gantry (Esaote G-Scan MRI system, Esaote S.p.A., Genoa, Italy)^22^. This system features a 0.25 T permanent magnet that can be rotated from 0° to 90° (see Fig. 1)—hence, it is further referred to as *rMRI*.

Standard Fourier transform image reconstruction of the Cartesian k-space data was applied for both systems using the vendor-supplied image processing units.

Only subjects with a body height below 175 cm, rather short thorax and a slim body form could be included in this study due to the space restrictions in the rMRI system and its limited field of view. All subjects met these inclusion criteria. They were placed first in an 80° upright standing position (see Fig. 1, right image). Again, a 3D localizer data set was first acquired to define a suitable image plane in the lung as described above. Then, dynamic imaging of a sagittal slice of the right lung with a 2D HYCE (bSSFP) sequence was performed with the following parameters: TR = 10 ms, TE = 5 ms, ST = 20 mm, Pixel Size 1.5 × 1.5 mm^2^, *α* = 80°. The temporal resolution was 1 fps. This measurement is later referred to as *upright rMRI.*

In 3 out of 8 subjects the patient bed of the *rMRI* system was rotated to the supine (0°) position, and the measurement was repeated (*supine rMRI*). As the same sequence parameters could be applied and the subject position was unchanged, the exact same slice could be imaged in upright and supine position. The slice position was verified with the help of fixed anatomic structures (e.g. distance of the measured slice to the spine). Results from *supine rMRI* could then also be used for comparison with data from *hMRI*. It could not be ensured, that the exact same cross-section was measured in both scanner systems due to the different field of view as well as temporal and special resolution. However, normalized movement curves could be compared to evaluate the general accordance of movement velocities during phonation in two different scanner systems.

The data sets from subjects 6 and 7 included only a reduced protocol (missing: VC breathing). As these measurements still met all other inclusion criteria and it was rather difficult to find adequate subjects due to the measurement procedure and the body configuration specifications for fitting in the *rMRI* system. These measurements were thus finally included in the analysis. A detailed description of which tasks were performed by which subjects can be seen in Supplementary Table S3 online.

### Audio recording

The audio signal was simultaneously recorded in the symphony scanner using a microphone system (Pre-polarized Free-field 1/2" Microphone, Type 4189, Brüel&Kjær, Nærum, Denmark) adapted for use in the MR environment. The length of the phonation was determined from the audio recording using Adobe Audition (CS6, Adobe systems Inc, San José, USA).

### MR image analysis

**Figure 2:**
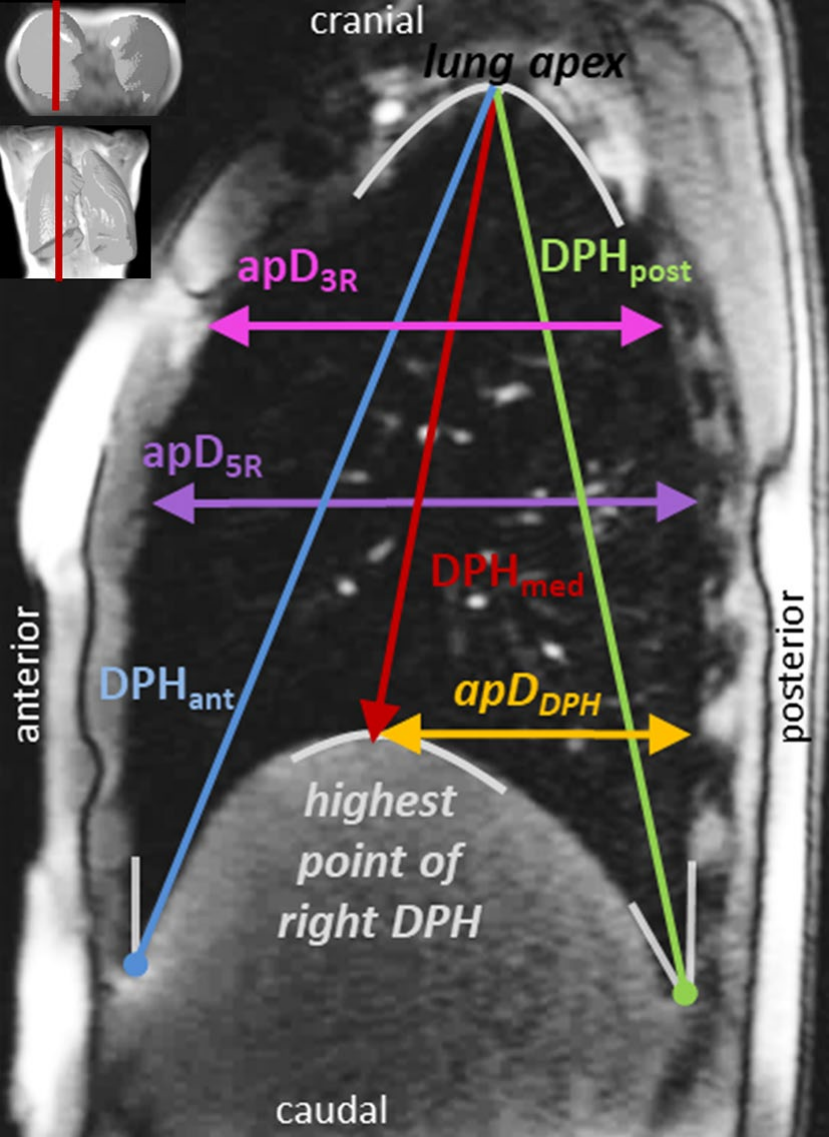
Measured distances in a sagittal plane of the right lung and their definition according to anatomical landmarks. Start and endpoints of measured distances according to anatomical landmarks (start–end). For details description see also Supplementary Table S3. DPH_ant_ = distance: anterior diaphragm–lung apex, DPH_med_ = distance: highest point of the diaphragm–lung apex, DPH_post_ = distance: posterior diaphragm–lung apex, apD_3R_ = horizontal anterior–posterior lung diameter: start of lung tissue–end of lung tissue at the height of the 3th rib, apD_5R_ = horizontal anterior–posterior lung diameter: start of lung tissue–end of lung tissue at the height of the 5th rib, apD_DPH_ = horizontal anterior–posterior diameter: highest point of the diaphragm cupola–end of lung tissue.

To characterize the motion of the breathing apparatus, distances between anatomical landmarks were manually measured in each acquired image frame (see Fig. 2 and Supplementary Table S4 online for details). The landmarks were chosen in accordance with a previous pilot study^8^. They are described in detail in Fig. 2 and Supplementary Table S3. Additionally to the pilot investigation^8^, the anterior–posterior (a-p) lung diameter at the height of the 3rd rib was measured to quantify the movement of the upper compartment of the thorax, and an a-p diameter from the highest point of the cupola of DPH to the posterior boundary of the lung was determined to detect a-p adjustments of the DPH.

Six different distance parameters were measured manually in each image, from which dynamic curves (8 subjects × 5 tasks × 6 parameters = 240 from supine hMRI and upright *rMRI* and 3 (subjects) × 5 (tasks) × 6 = 90 from the supine *rMRI*) were created. One image sequence (subject 3, A3, upright) was measured twice by one rater and additionally by another second rater to allow for calculation of intra- and inter-rater reliability.

### Time and amplitude normalisation

As thorax sizes and pitch durations differed for the volunteers, parameter curves could not be directly compared. Therefore, the time of each movement curve (*t*_norm_) was renormalized starting at the beginning of phonation (*t*_start_) and ending with end of phonation (*t*_end_). Additionally, distances were normalized (*A*_norm_) to the amplitude at the start (*t*_start_) and the end (*t*_end_) of phonation:

$$A_{norm} \left( t \right) = \frac{{A\left( t \right) - A\left( {t_{end} } \right)}}{{A\left( {t_{start} } \right) - A\left( {t_{end} } \right)}} \cdot 100$$

Then, the temporal derivative *m*(*t*) was calculated for all normalized curves at $n$ = 5 equidistant times along the normalized time axis for statistical evaluation in 20% timesteps (*m*_1_ to *m*_5_):

$$m_{n} = m(t_{n} ) = \frac{{\Delta A_{norm} }}{\Delta t} = \frac{{A_{norm} \left( {t_{n} } \right) - A_{norm} \left( {t_{n - 1} } \right)}}{{\left( {t_{n} - t_{n - 1} } \right)}}$$

**Figure 3:**
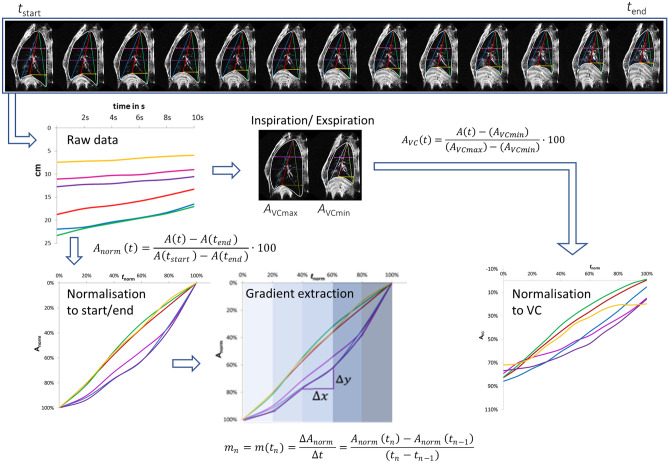
Flow-chart of data normalisation and analysis. Raw data of 6 distance parameters were measured in each acquired image sequence. For inter-individual comparison. The timeline has been rescaled: The start of phonation was set to 0% the end point to 100%. Additionally, the distance parameters were normalized to start and end point of phonation (left part) as well as to maximum inspiration and expiration (right part). VC stands for vital capacity.

To allow for comparison of movement curves of the respiratory apparatus also in relation to subjects’ individual vital capacity an additional amplitude normalization (A_VC_) was performed: Here the amplitude at maximal inspiration (A_VCmax_) was set to 100% and the amplitude at maximal expiration (A_VCmin_) was set to 0%. Time was scaled as described above. This was not done for subjects 6 and 7 due to the lack of VC breathing data. The process of normalisation is also visualized in Fig. 3.

From the raw movement curves the covered distance for each measured parameter during phonation was also derived as an individual motion range (A_∆Phon_) as A_∆Phon_ = A(t_start_) − A(t_end_). As comparison of raw data is not expedient due to inter-individual differences in dimensions of the respiratory system it was then normalized in relation to A(t_start_) for each location as A_∆Phon%_ = A_∆Phon_ * 100/A(t_start_)).

### Statistical analysis

In a first step intraclass correlation coefficient (= ICC) was calculated to evaluate intra- and inter-rater reliability. Double measurements were made for one image sequence (subject 3, P1mf upright).

The time derivatives ${m}_{n}$ were statistically analysed in 20% steps using repeated-measures ANOVA that compares means across all variables based on repeated observations in *m*_*1*_ to *m*_*5*_. (= factor 5*).* To control for confounding variables, task and location were regarded as covariates.

Differences in phonation time for different tasks or individual movement ranges between different body positions were calculated using an univariate ANOVA. Statistically significant differences were further analysed using Tukey's-HSD post-hoc test.

For all statistical analyses, SPSS (SPSS Inc. SPSS for Windows, Version 27.0, Chicago, IL) was used. The level of significance was set to *p* < 0.05.

## Results

The ICC was calculated to estimate intra- and inter-rater reliability. The ICC between two different raters calculated over all parameters for one image sequence (subject 3, P1mf upright) was ICC = .996 (all locations separately are displayed in supplement table S5). The ICC for intra-rater reliability was ICC = .998 (all locations separately are displayed in supplement table S5).

Respiratory movements during phonation were recorded with two different MR scanners. Therefore, in the first step, the concordance of the data of different sessions was verified comparing supine data in both scanners.

### Supine *rMRI* versus supine *hMRI*

**Figure 4:**
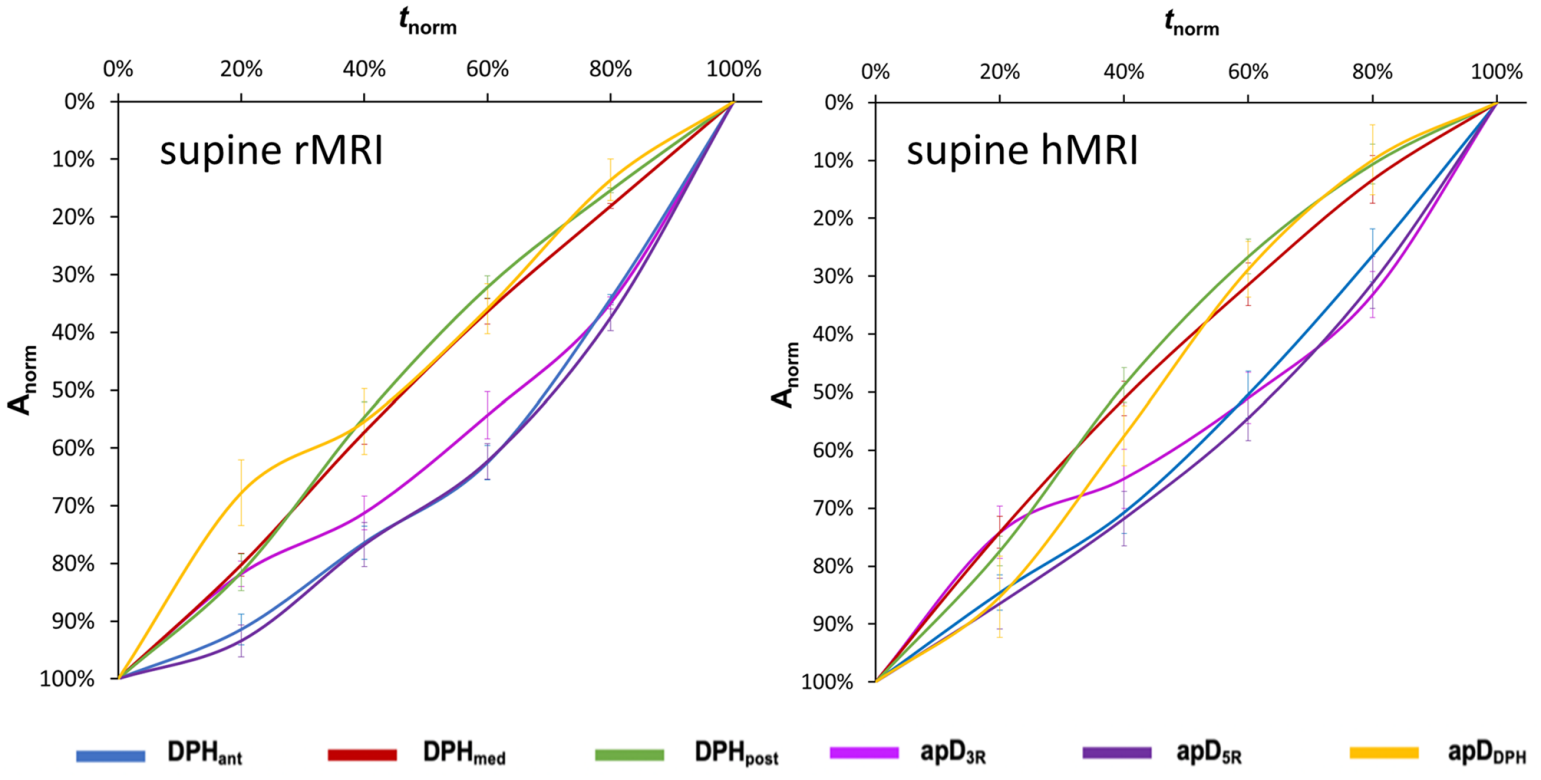
General agreement of normalized motion curves from rotatable MRI (= rMRI) and conventional MRI (hMRI) data in supine position as error detection experiment. Normalized motion curves of 6 measured distance parameters from dynamic MRI acquired in supine body position in hMRI and rMRI in subjects 1–3. Normalized time is displayed on x-axis, mean normalized amplitude with standard deviation on y-axis. Different locations are displayed in different colours.

Supine measures from both scanners were compared in the first step as an error detection experiment. Mean maximum phonation time did not differ significantly between supine *rMRI* (mean: 13.2 s) and *hMRI* (15.8 s) (F(1/29) = 1.48, *p* = .23, ƞ^2^ = .05). In Fig. 4 no major difference can be seen in the normalized motion curves between supine rMRI and hMRI. Additionally, statistical evaluation of the time-derivatives with repeated measures ANOVA for factor scanner (covariates: task and location) showed no significant difference in the overall curve progression (F(4/172) = 1.72, *p* = .15, ƞ^2^ = .03). Still, a differentiated calculation for all locations separately revealed that the curve progression of the anterior–posterior position of the DPH (apD_DPH_) behaved differently while no difference was found for all other locations (see Supplementary Table S6).

### Upright *rMRI* versus supine *hMRI*

**Figure 5:**
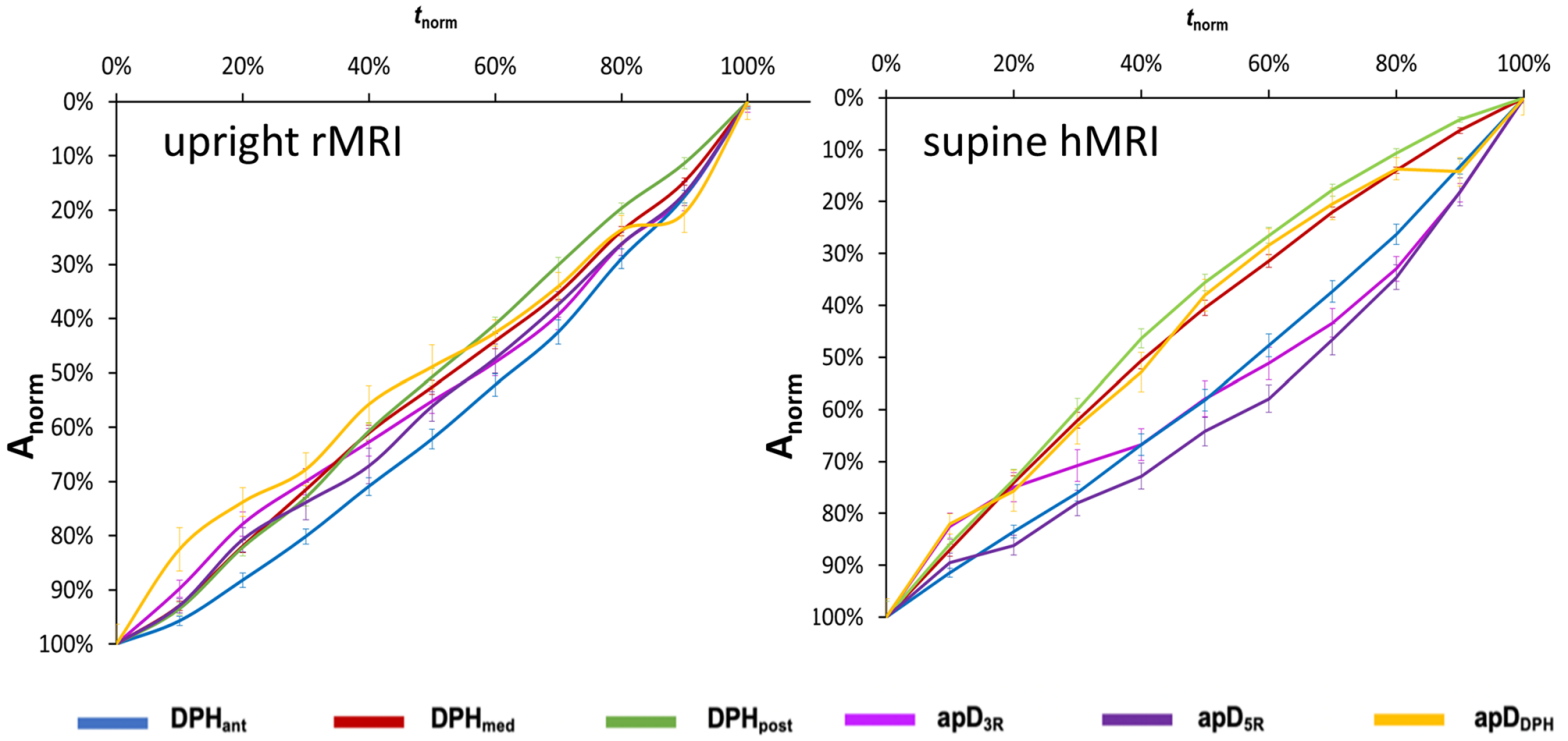
Elliptic curve shape with two different motion patterns in supine phonation (right diagram) is less pronounced in upright phonation (left diagram). Upright rMRI data is displayed on the left, supine hMRI data on the right. Mean normalized curve amplitude (A_norm_) and standard error is displayed on the y-axis and normalized time (t_norm_) is displayed on the x-axis. Different locations are displayed in different colours.

Upright *rMRI* and supine *hMRI* data were then compared in all subjects. Duration of sustained phonation did not differ significantly between upright and supine (mean upright maximum phonation time = 17.6 s, mean supine maximum phonation time = 18.5 s; F(1/71) = .28; *p* = .60, ƞ^2^ = .003). Curves of supine data (see Fig. 5, right diagram) can be differentiated in two different movement velocity patterns resulting in an elliptic shape of curves: The first is characterised by a quicker movement velocity at the beginning which slows at the end. The second shows the inverse with a slower movement velocity at the beginning which quickens at the end. The fist pattern was found for the medial (DPH_med_)/ posterior diaphragm (DPH_post_) and diaphragm cupola (apD_DPH_) and the second was found for the anterior diaphragm (DPH_ant_) and both rip cage parameters (apD_5R_, apD_3R_). This difference is visually less pronounced in upright body position (see Fig. 5 left diagram). Here the curve progression seems more linear. Still, general statistical evaluation of curve progression between upright and supine phonation showed no significant difference (F(4/424) = 1.70, *p* = .15, ƞ^2^ = .18). A further calculation for all locations separately could still underline visual differences between motion curves of upright and supine phonation for the DPH_med_, apD_5R_ and apD_DPH_ (Supplementary Table S7). These curves were significantly more linear in the upright phonation compared to the more differentiated motion pattern in supine phonation.

No statistical difference in curve gradients was found concerning different tasks for either the upright nor supine data: supine: F(4/209) = 1.33, *p* = .17, ƞ^2^ = .03; upright: F(4/209) = .93, *p* = .54, ƞ^2^ = .02, for separate calculation of all locations see Supplementary Table S8 and Supplementary Fig. S12).

### Phonatory movement ranges from upright rMRI versus supine hMRI

**Figure 6:**
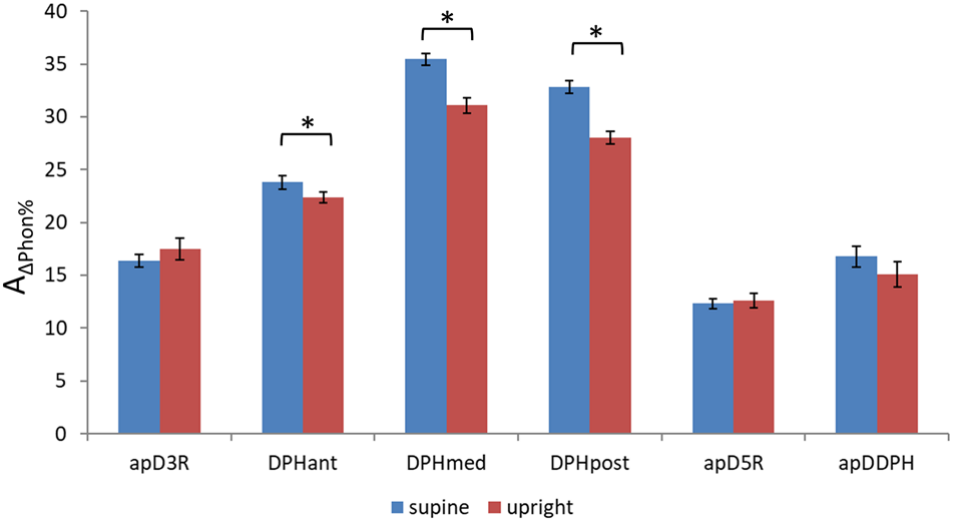
Larger cranial diaphragm displacement during supine compared to upright phonation. Y-axis shows the percentual movement range (A_∆Phon%_) during phonation. Bars represent different locations in upright (red) and supine (blue) position including standard error. Significant differences are marked with *. All p-values are displayed in Supplementary Table S9 online.

The distances covered by different parts of the respiratory system during phonation were quantified as A(t_start_) − A(t_end_) and inter-individually compared in relation to the starting position, later referred to as percentage movement range (A_∆Phon%_). Figure 6 displays mean values of A_∆Phon%_ for all locations. Visual analysis shows that DPH displacement during phonation was pronounced in the supine position while the posture related difference in the thorax movement was minor. An additional statistical evaluation confirmed that the amount of DPH elevation during phonation was larger in the supine compared to the upright body position. All *p*-values and effect sizes are displayed in Supplementary Table S9 online.

### Respiratory normalized data from upright rMRI versus supine hMRI

**Figure 7:**
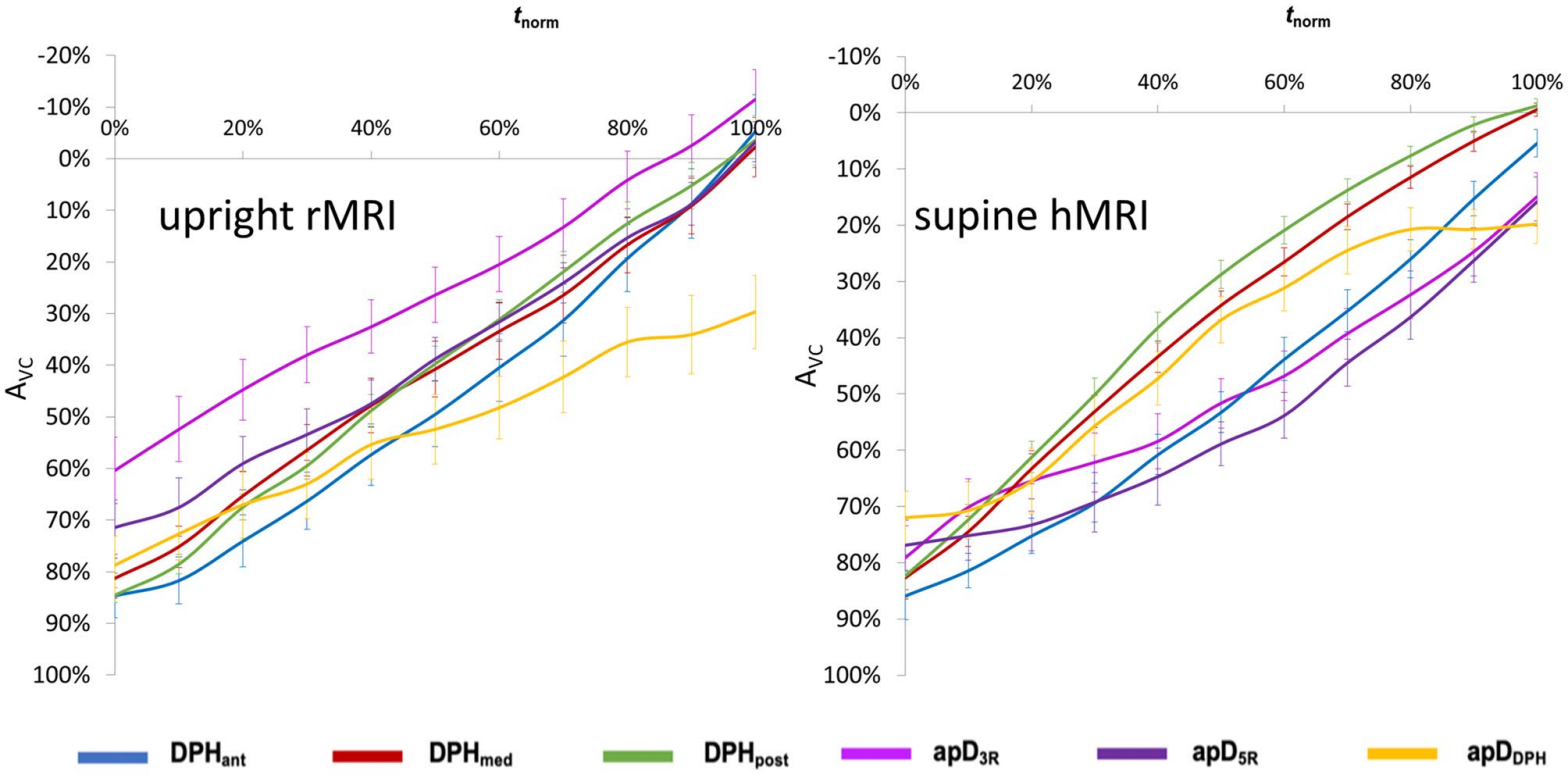
Thoracic movements during phonation take place at 20% higher vital capacity range in supine position. Upright rMRI data is displayed on the left, supine hMRI data on the right. Normalized curve amplitude according to individual maximum inspiration and expiration (AVC) is displayed on y-axis and normalized time (t_norm_) is displayed on x-axis. Different locations are displayed in different colours.

As explained in detail in the method section, to differentiate active phonatory compensations from sole respiratory adaptations the amplitude was additionally normalized according to maximal inspiration and maximal expiration. In general, subjects started their phonation in a supine position at a higher lung volume in relation to respiration (mean start supine: 81.72% of VC versus mean start upright = 75.86% of VC; F(1/356) = .07, *p* = .02, ƞ^2^ = .013). They also stopped phonating at a higher lung volume in the supine compared to upright body position (mean end supine 9.15% of VC, mean end upright = − 1.30% of VC, F(1/356) = 13.42, *p* < .001, ƞ^2^ = .03). A differentiated view on the six movement parameters in Fig. 7 reveals that especially the thorax was expanded more during inspiration for phonation in the supine compared to upright position. However, its movement also terminated at a higher lung volume. In accordance with these results, the thorax was less expanded during inspiration for phonation in upright position and also the lowering of the thorax during phonation terminated at a lower lung volume, with even negative values observed especially for the upper thorax. This observation is confirmed statistically for the upper thorax which increased in diameter from 58.25% of VC inspiration in the upright to 79.11% in the supine position (F(1/59 = 5.93, *p* = .02, ƞ^2^ = .09). For all other locations this difference did not reach statistical significance (for all p-values see Supplementary Table S10). The upper and lower thorax diameters also terminated their phonatory movement with a significant posture related difference at 20–30% higher lung volume in the supine position (apD_3R_: F(1/59) = 16.88, *p* < .001, ƞ^2^ = .23, apD_5R_: F(1/59) = 12.59, *p* = .001, ƞ^2^ = .18, for all p-values see Supplementary Table S10). The mean delta of the phonatory movement did not differ significantly (F(1/356) = 1.79, *p* = .18, ƞ^2^ = .005) between upright and supine phonation. While the thorax position during phonation differed significantly from the VC respiration between upright and supine phonation, posture related differences in DPH position in relation to breathing were minor during sustained phonation.

### Upright versus supine rMRI

**Figure 8:**
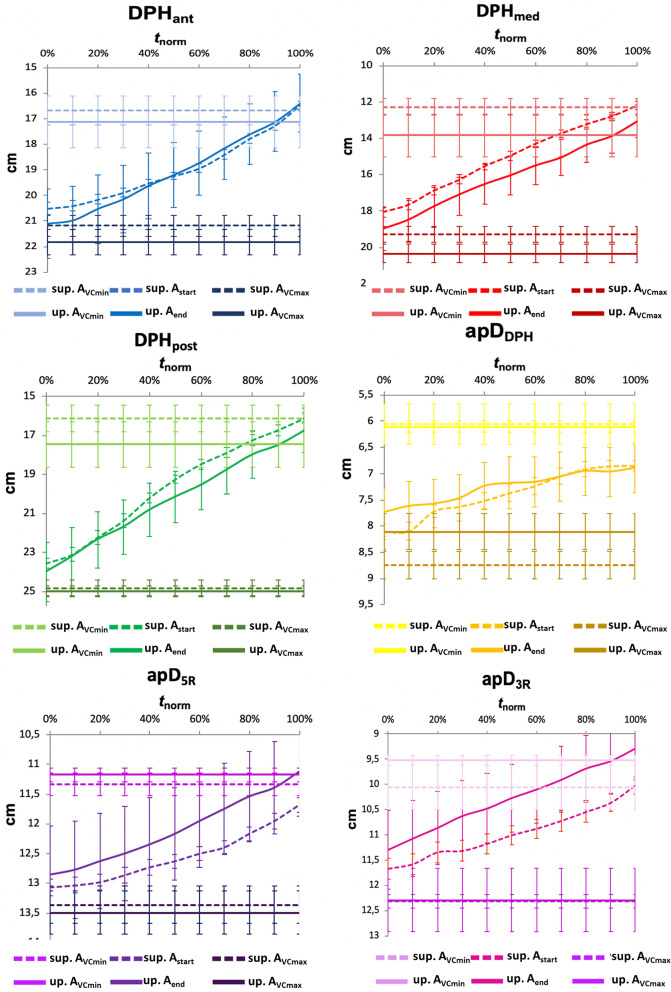
Cranial displacement of diaphragm motions during breathing and phonation; larger rip cage diameters during phonation in supine body position. Mean raw data for three subjects with standard error in cm is displayed on the y-axis, normalized time on the x-axis for each location. Dashed lines represent supine, solid lines upright data. Different shades of colour represent vital capacity inspiration/ expiration and sustained phonation. For better comparability, vital capacity maximum inspiration and expiration are included as horizontal lines.

Additionally, raw data of the three subjects for whom both upright and supine rMRI data was collected (subject 1–3) was compared. For these subjects an additional measurement was possible in the rMRI in supine position. Using anatomical landmarks in the same field of view in the same scanner enabled comparison of the exact same cross-sectional plane and therefore raw data without normalisation. In the supine position, the diaphragm was more cranially positioned during maximum inspiration and expiration compared to the upright position (see Fig. 8). During vital capacity inspiration, while the upper thorax was placed outwards in the supine rather than upright position, there was little difference observed for the lower thorax. The DPH cupola was positioned more anteriorly during maximum inspiration (apD_DPH_) in the supine compared to upright position.

**Figure 9:**
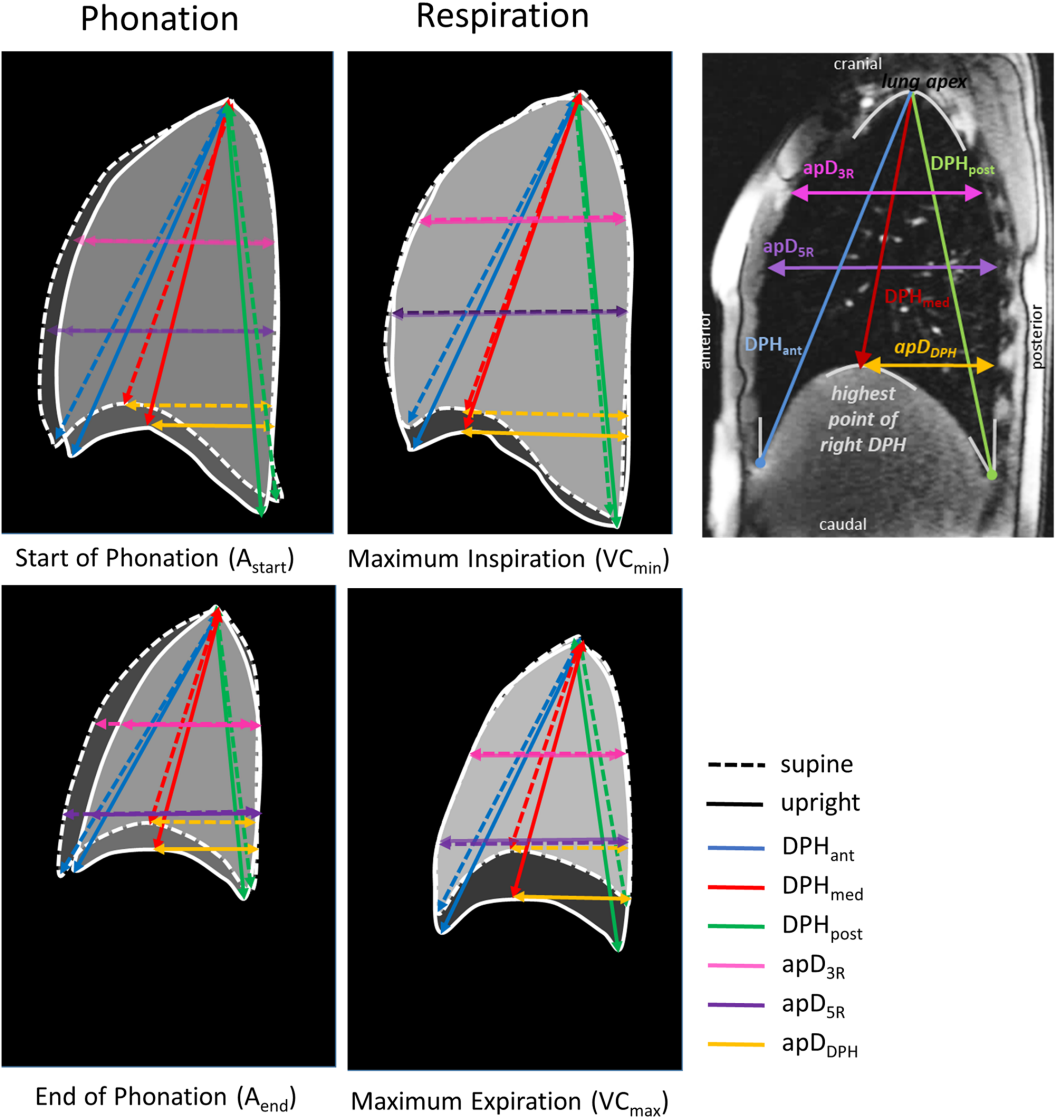
Exemplary visualisation of differences in lung dimensions of subject 3 derived from MRI data at the start and end of phonation of A4 as well as maximum inspiration and expiration in the upright and supine positions. The dimensions of the right lung in sagittal cross-section are shown as a grey area on a black background and differentiated for the upright and supine body positions based on the outline (dashed = supine, solid = upright). Measured distance parameters are displayed in different colours according to Fig. 2.

In the supine position, the DPH was also more cranially positioned and to the same extent during both phonation and breathing. Thus, its movement took place at lower VC levels. These differences were pronounced in the medium part of the DPH. In contrast, the rip cage diameter showed a strong tendency to be larger in the supine position and its movement took place at higher VC levels. These differences are observable exemplarily for subject 3 in Fig. 9 and Supplementary Video S11 online with phonation at pitch A4 and respiration in upright versus supine body position.

## Discussion

This study presents an MRI-based analysis of postural changes in respiratory kinematics during phonation in professionally trained singers. Increased inspiratory thoracic elevation in the supine position allows professional singers to maintain their general breathing strategies with “mixed” inspiratory slowing of the respiratory movement for sustained phonation regardless of body position.

In the acquired images six distances were determined and normalized for the motion analysis. A linear transglottal airflow and, thus, pulmonary ventilation is needed for sustained phonation^23^. The general breathing strategy of professional singers to accomplish this task is characterized by an elliptical curve shape of these measures (cf. Figure 5) as described previously^8^. This shape originates from two velocity groups: At the beginning of the phonation of a sustained tone, the lung has a high volume with a need for pressure reduction as expiratory elastic recoil forces exceed the intended subglottic pressure. The inspiratory thorax muscles and the anterior DPH are activated here leading to a slowing of the respiratory movement in these parts of the breathing apparatus. Simultaneously, the medial and the posterior part of the DPH relax increasingly and are thus cranially displaced at a higher movement velocity. This braking phase is followed by a reduction of inspiratory activation that is precisely adjusted to the decrease of passive expiratory recoil forces until the intended subglottic pressure is reached. To maintain phonation at lower lung volumes, the singer needs to exert additional expiratory forces onto the lung, as the inspiratory recoil forces from the elastic properties of the thorax and the lung increase. Expiratory muscles of the thorax and the abdominal wall are thus activated, resulting in higher movement velocities in the thorax and anterior DPH but also a slower DPH elevation in the back part (where the possible range of motion is already exhausted). The phonation stops when inspiratory recoil forces exceed the expiratory forces. In both a previous^8^ and this study, no large effect of pitch or loudness was found on this movement pattern. But, these parameters had an effect on the length of phonation, which was shorter for higher pitched phonation^8^. A similar motion pattern was also described by Hixon et al. for upright phonation of sustained speech utterances in untrained subjects^7,15^. However, unlike the data presented here, their assumptions were based on measurements of the thorax and abdomen diameter by magnetometry rather than imaging. The movement of the diaphragm often correlates with the movement of the abdomen, but it is also dependent on the degree of tension of the abdominal muscles, which was not recorded here.

Comparison of normalized motion curves in upright and supine body positions showed that the previously described strategy could generally be maintained in both body positions. However, the differentiation in two velocity groups was more pronounced in the supine position and the movement sequence was more linear in the upright position especially in the mid part of the DPH as well as the thorax diameter at the height of the 5^th^ rip (cf. Figure 5). This is physiologically plausible, since in the supine position a higher expiratory force acts on the respiratory system and therefore requires a greater inspiratory slowing of the respiratory movement, especially at the beginning of phonation. The thorax and the anterior DPH primarily slow the respiratory movement to prevent the direct transfer of the additional passive expiratory forces to the subglottic pressure. In contrast to these results, a fundamental change in the respiratory kinematics during supine phonation was described in untrained subjects^15^: The group of Hixon et al. described that untrained subjects performed the inspiratory retardation of the respiratory movement only with their diaphragm while the thorax muscles did not contribute to breath support in supine position. Still, the DPH movement was only indirectly measured in this study, which used magnetometry instead of imaging. Still, Hixon et al. postulated, that using the DPH for inspiratory slowing of the respiratory movement is “the only efficient strategy for counteracting the stronger relaxation pressure at large lung volumes in the supine body position because the major component of relaxation pressure comes from the abdomen”^15^. However, maintaining the mixed inspiratory strategy despite changes in body position is only possible if active compensatory movements of the respiratory muscles balance the gravitational forces. For this purpose singers adapted both the thorax and DPH positions for phonation:

Professional singers expanded their chests by an additional 20% during inspiration when singing in the supine position. In addition, the thoracic movements then took place at 20% higher vital capacity range in the supine position but its movement range did not increase. At first glance, this difference seems inconsequential, since the expiratory effect of gravity on the thorax is increased in the supine position compared with the upright body position. Therefore, one might expect a gravitational induced reduction in thorax width. The results also contrast those of untrained subjects’ data from running speech where volume events in the supine body position occured at 20% lower lung volume^7^. Interestingly the extra thorax inspiration was pronounced during phonation but not during sole respiration, thus this compensation could be related to the specific requirement on breath support during phonation. When singers out-perform the expiratory forces of gravity through increased inspiration, inspiratory slowing of thoracic lowering during phonation is once again reasonable for subglottic pressure regulation. Thus, this compensatory mechanism may be the key point in the maintenance of the mixed breath support strategy in singers. Since the singers were not actively instructed to do this, but rather responded intuitively in this way, it seems to be beneficial for the quality of singing to not fundamentally alter the breath support function when in a supine position. There is also evidence from previous studies showing that a change in habitual breathing kinematics during singing can lead to a poorer sonic outcomes^24^. It is known that while singers can largely level out the effects of lung volume via tracheal pull on the laryngeal position through training, these interactions are more pronounced in the untrained^25–27^. When singers were forced to abandon their habitual respiratory behaviour, their voice function was again more strongly influenced by lung volume, similar to the relations found for vocally untrained subjects^28^.

In contrast to this inspiratory shift of thorax movement during phonation, the DPH was cranially/ expiratory displaced in the supine position by an average of 1 cm in both breathing and phonation. In a supine position gravity acts on an expiratory direction on the abdomen, which leads to an increased abdominal pressure on the DPH. The singers could not fully compensate for the related cranial displacement of the diaphragm. But still, the DPH cupola (apD_DPH_) was positioned more anteriorly during maximum inspiration and phonation in the supine position which is typically associated with an increase in contraction force probably to compensate for gravitational effects. Sundberg et al. also found a more forceful contraction of the DPH during inhalation in supine position^14^. During singing, however, the diaphragm was then displaced more strongly in the cranial/expiratory direction, thereby increasing its range of motion. This led to a more effective squeezing of the lungs at the end of phonation with reduction of the expiratory reserve volume. Previous studies had already shown the amplification of diaphragmatic movements during respiration^13^.

While the effects of gravity on the functional unit of the diaphragm and abdomen and its compensatory strategies were uniform in direction and magnitude for respiration and phonation, the increase in thoracic volume was specific for phonation. Interestingly former studies found that singers could control their vocal tract configuration very precisely regardless of body position except for the vertical larynx position which was also cranially displaced in supine singing^16^. As mentioned above, the respiratory movements are closely related to vertical laryngeal position mediated by the tracheal pull^29^. As singers did not fully compensate for the gravity related cranial displacement of DPH for supine phonation this would in turn reduce the tracheal pull and explain the more cranial position of the larynx in supine singing. The knowledge of these interactions, as well as the position-dependent compensatory movements described, could also be applied in voice pedagogy and voice therapy. For example, singing styles that often show more thorax-bound breathing kinematics^30^ and higher larynx position^31^, such as belting, could be trained more in the supine position. Comparative singing in upright and supine body positions could be used to become more aware of potential changes in respiratory movements and laryngeal positioning.

Professionally trained singers can repetitively generate consistent and uniform breathing movements^2,18,19,32–35^. In this study, all the professional singers were familiar with singing in different body positions, as a requirement in a modern director’s theatre. Thus, these volunteers are best suited for a comparison of phonation in different body positions–nevertheless, the precise uniform execution of the respiratory movement during singing cannot be verified for each task, which is a limitation of this study. To minimize this effect, the subjects and the instructors actively selected only those data for further analysis in which the voice quality and pitch met the requirements of fundamental frequency and sound pressure level.

Data was acquired with two MR systems (rMRI: upright and supine, hMRI: supine) with different field strengths as well as temporal and spatial resolution. Nevertheless, a general agreement of the motion data from both scanners was shown in a subgroup analysis for most of the parameters. For this purpose, data recorded in the supine position from both scanners were analysed. In this subgroup the maximum phonation time was similar, which indicates similar measurement conditions. A direct image comparison was not possible as scanner-related slightly different image locations were chosen. All data normalized in amplitude and time showed no statistical differences between the movement curves in both MR systems in supine position (cf. Fig. 4). Further analysation of all parameters individually revealed a significant difference in the curve progression for apD_DPH_. Here especially in the first section the DPH moved quicker backwards in the rMRI compared to hMRI. Whether this difference is due to a certain variability in the DPH movement or arose due to the possibly slightly different imaged slice can unfortunately not be differentiated conclusively. Overall, however, a very high intra- and inter-rater reliability was shown, so that differences are rather not due to the process of measurement.

For statistical analysis pooled data of all subjects and tasks were used. Analysis was performed with repeated measures ANOVAs and inclusion of covariates controlled for confounding variables. This statistical approach violates the independence of observations, but the limited availability of professional trained singers willing to perform in a tiltable MRI system justifies this approach in the authors’ opinion. In the authors' view, the advantage of including multiple tasks in the analysis, rather than a single pitch, lies in the more comprehensive reflection of the singers' phonation. When the tasks were designed, it was not clear to what extent different pitches and volumes differed in respiratory kinematics. However, the present data (Supplementary Table S8 and Supplementary Figure S12) as well as the data from the pilot study^8^ do not show a large influence of pitch or volume. The different tasks were therefore statistically considered as repeat measurements.

Thus, the statistical results only provide a first estimate and should be verified in a larger cohort. Finally, in the noisy environment of an MRI system the Lombard effect on phonation^36^ cannot be excluded even for professionally trained singers—however, as data were acquired in the MRI only, this effect should be cancelled to first order.

## Conclusion

In previous studies of untrained subjects posture related changes in respiratory function for phonation have been postulated^15^ that are inconsistent with results in this study of professional singers. Professional singers (over)compensated gravitation-related expiratory effects on the thorax by increased inspiration and thus maintained their general concept of breath support regardless of the body position. The DPH was cranially displaced but its movement range increased for supine phonation. While the effects of gravity on the functional unit of the diaphragm and abdomen and its compensatory strategies are uniform in direction and magnitude for respiration and phonation, the overcompensation of thoracic inspiration was more specific for phonation. These results can inform a more realistic extrapolation of research results obtained in the supine position and could be used to train specific respiratory strategies for phonation.

## Supplementary Information

Supplementary Tables.

Supplementary Video 1.

Supplementary Figure 1.
