# Supplementary material for: The influence of gravity on respiratory kinematics during phonation measured by dynamic magnetic resonance imaging
Source: Sci Rep. 2021 Nov 25;11:22965. doi: 10.1038/s41598-021-02152-y (PMC8617256; doi:10.1038/s41598-021-02152-y)
Supplement: Supplementary file 1 — Supplementary Tables. [file 41598_2021_2152_MOESM1_ESM.docx]

# Supporting Information

**Supplementary Table S1: Subject number, age, gender, voice classification, classification according to the Bunch and Chapman** (1) **taxonomy, vital capacity (=VC), forced expiratory volume in one second (= FEV1), body height and weight.**

| Subject | Age | Gender | Voice Classifi-cation | Bunch/ Chapman taxonomy | VC in l  supine | VC in l upright | FEV1 in l/s  upright | Height in cm | Weight in kg |
| --- | --- | --- | --- | --- | --- | --- | --- | --- | --- |
| 1 | 25 | female | soprano | 3.15b1 | 3.04 | 3.87 | 3.46 | 167 | 55 |
| 2 | 28 | female | soprano | 4.5 | 3.24 | 4.72 | 3.88 | 165 | 65 |
| 3 | 25 | female | soprano | 3.15b1 | 3.41 | 3.71 | 3.28 | 158 | 47 |
| 4 | 34 | male | tenor | 3.4 | 3.82 | 4.40 | 3.35 | 175 | 66 |
| 5 | 32 | female | soprano | 3.1b | 3.27 | 3.28 | 2.90 | 157 | 53 |
| 6 | 33 | male | tenor | 5.4 | 4.45 | 4.92 | 3.21 | 171 | 62 |
| 7 | 32 | female | soprano | 5.4 | 4.56 | 4.72 | 3.77 | 170 | 65 |
| 8 | 27 | male | tenor | 7.1 | 5.13 | 5.28 | 4.11 | 172 | 80 |

**Supplementary Table S2:** **Description of the tasks performed by subjects according to different voice classifications including pitch and loudness. mf = mezzo forte, ff = fortissimo, pp = pianissimo**

| Voice classification: | Soprano | | Tenor | |
| --- | --- | --- | --- | --- |
| Task: | pitch | loudness | Pitch | loudness |
| P1mf: | A3 (220Hz) | mf | A2 (110Hz) | mf |
| P2mf: | A4 (440Hz) | mf | A3 (220Hz) | mf |
| P3mf: | A5 (880Hz) | mf | A4 (440Hz) | mf |
| P2pp: | A4 (440Hz) | pp | A3 (220Hz) | pp |
| P2ff: | A4 (440Hz) | ff | A3 (220Hz) | ff |

**Supplementary Table S3:** **Description of which tasks were performed by which subjects. Measured data is marked in green and missing data in red.**

MRI

Subject

|  | **upright rMRI** | | **supine rMRI** | | **hMRI** | |
| --- | --- | --- | --- | --- | --- | --- |
|  | **breathing** | **phonation** | **breathing** | **phonation** | **breathing** | **phonation** |
| 1 |  |  |  |  |  |  |
| 2 |  |  |  |  |  |  |
| 3 |  |  |  |  |  |  |
| 4 |  |  |  |  |  |  |
| 5 |  |  |  |  |  |  |
| 6 |  |  |  |  |  |  |
| 7 |  |  |  |  |  |  |
| 8 |  |  |  |  |  |  |

**Supplementary Table S4: Anatomical definition of the 6 locations for measured distances parameters.**

| **Sagittal plane** | |
| --- | --- |
| **DPH_ant_** | Craniocaudal lung height from the angle of the anterior DPH and the RC to the apex of the lung |
| **DPH_med_** | Craniocaudal lung height from highest point of DPH to the apex of the lung |
| **DPH_post_** | Craniocaudal lung height from the angle of the posterior DPH and the RC to the apex of the lung |
| **apD_3R_** | the anterior-posterior lung diameter at the height of the 3th rib |
| **apD5_R_** | the anterior-posterior lung diameter at the height of the 5th rib |
| **apD_DPH_** | the anterior-posterior diameter from the highest point of the cupola of the DPH to the posterior boundary of the lung |

**Supplementary Table S5: Interclass correlation coefficient (ICC) for inter- and intra-rater reliability for all locations seperately as defined in Fig. 2 and Supplementary Table S4.**

|  | DPH_ant_ | DPH_med_ | DPH_post_ | apD_3R_ | apD_5R_ | apD_DPH_ |
| --- | --- | --- | --- | --- | --- | --- |
| ICC for inter-rater reliability | **.978** | **.971** | **.986** | **.782** | **.987** | **.958** |
| ICC for intra-rater reliability | **.980** | **.995** | **.982** | **.997** | **.991** | **.931** |

**Supplementary Table S6: Position-related difference of curve progression for supine rMRI vs. supine hMRI, calculated for all locations separately as defined in Fig. 2 and Supplementary Table S4.**

|  | DPH_ant_ | DPH_med_ | DPH_post_ | apD_3R_ | apD_5R_ | apD_DPH_ |
| --- | --- | --- | --- | --- | --- | --- |
| Supine rMRI vs.  Supine hMRI | F(4/70) = 2.10  p = .09  ƞ^2^ = .07 | F(4/70) = 1.00  p = .40  ƞ^2^ = .03 | F(4/70) = .27  p = .85  ƞ^2^ = .01 | F(4/70) = .33  p = .81  ƞ^2^ = .01 | F(4/70) = .90  p = .46  ƞ^2^ = .03 | F(4/70) = 6.30  P< .001  ƞ^2^ = .18 |

**Supplementary Table S7: Position-related difference of curve progression for upright rMRI vs. supine hMRI, calculated for all locations separately** **as defined in Fig. 2 and Supplementary Table S4.**

|  | DPH_ant_ | DPH_med_ | DPH_post_ | apD_3R_ | apD_5R_ | apD_DPH_ |
| --- | --- | --- | --- | --- | --- | --- |
| Upright rMRI vs.  Supine hMRI | F(4/395) = 2.44  p = .06  ƞ^2^ = .03 | F(4/395) = 9.80  p < .001  ƞ^2^ = .12 | F(4/395) = .60  p = .82  ƞ^2^ = .08 | F(4/395) = 1.58  p = .20  ƞ^2^ = .02 | F(4/395) = 2.92  p = .03  ƞ^2^ = .04 | F(4/395) = 2.95  p= .038  ƞ^2^ = .04 |

**Supplementary Table S8: Task-related difference of curve progression for upright rMRI and supine hMRI, calculated for all locations separately as defined in Fig. 2 and Supplementary Table S4.**

|  | DPH_ant_ | DPH_med_ | DPH_post_ | apD_3R_ | apD_5R_ | apD_DPH_ |
| --- | --- | --- | --- | --- | --- | --- |
| Upright rMRI | F(4/195) = .65  p = .80  ƞ^2^ = .09 | F(4/195) = .40  p = .95  ƞ^2^ = .06 | F(4/195) = .60  p = .82  ƞ^2^ = .08 | F(4/195) = 1.38  p = .20  ƞ^2^ = .12 | F(4/195) = 1.4  p = .12  ƞ^2^ = .18 | F(4/195) = .97  p= .48  ƞ^2^ = .13 |
| Supine hMRI | F(4/195) = .56  p = .87  ƞ^2^ = .08 | F(4/195) = 1.11  p = .37  ƞ^2^ = .13 | F(4/195) = 1.70  p = .06*  ƞ^2^ = .22 | F(4/195) = 1.17;  p = .32  ƞ^2^ = .15 | F(4/195) = .90  p = .56  ƞ^2^ = .15 | F(4/195) = 1.20  p = .29  ƞ^2^ = .15 |

**Supplementary Table S9:** **Position-related difference of individual movement ranges during phonation (A_∆Phon%_) individually calculated for all locations** **as defined in Fig. 2 and Supplementary Table S4.**

|  | DPH_ant_ | DPH_med_ | DPH_post_ | apD_3R_ | apD_5R_ | apD_DPH_ |
| --- | --- | --- | --- | --- | --- | --- |
| A_∆Phon%_ upright vs. supine | F(1/69) = 7.58,  p = .008  ƞ^2^ = .10 | F(1/69) = 17.30,  p <.001  ƞ^2^ = .18 | F(1/69) = 19.10,  p <.001  ƞ^2^ = 2.20 | F(1/69) = .71, p = .40  ƞ^2^ =.01 | F(1/69) = .13, p = .72  ƞ^2^ = .002 | F(1/69) = 1.19,  p = .28  ƞ^2^ = .02 |

**Supplementary Table S10:** **Start and end point of phonation in relation to vital capacity (VC) breathing and results of statistical evaluation between upright and supine body position.**

|  | **Start at % of VC supine** | **Start at % of VC upright** | **Difference between uptight and supine** | **End at % of VC supine** | **End at % of VC upright** | **Difference between upright and supine** |
| --- | --- | --- | --- | --- | --- | --- |
| **DPH_ant_** | 89.74% | 84.13% | F(1/59) = 1.11, p = .23, ƞ^2^ = .02 | 5.55% | - 7.77% | F(1/59) = 3.29, p =.,08, ƞ^2^ = .05 |
| **DPH_med_** | 85.30% | 80.66% | F(1/59) = .99, p = .32, ƞ^2^ = .02 | - .85% | - 3.74% | F(1/59) = .26, p = .61, ƞ^2^ = .004 |
| **DPH_post_** | 84.22% | 84.04% | F(1/59) = .01, p = .95, ƞ^2^ = <.001 | - 1.55% | - 4.53% | F(1/59) = .38, p = .54, ƞ^2^ = .006 |
| **apD_3R_** | 79.11% | 58.25% | F(1/59) = 5.93, p = .02, ƞ^2^ = .09 | 16.43% | -12.70 % | F(1/59) = 16.88, p < .001, ƞ^2^ = .23 |
| **apD_5R_** | 77.10% | 68.49% | F(1/59) = 1.70, p = .20, ƞ^2^ = .03 | 15.70% | -4.75% | F(1/59) = 12.59, p = .001, ƞ^2^ = .18 |
| **apD_DPH_** | 74.84% | 78.68% | F(1/59) = .32, p = .58, ƞ^2^ = .01 | 19.61% | 32.68% | F(1/59) = 2.68, p = .11, ƞ^2^ = .04 |

**Supplementary Video S11: Exemplary representation of the measured distances during respiratory movement for the sustained phonation of A4 (440Hz) sung by subject 3 (female) in upright and supine position in the rotatable MRI.** Left image represents supine, right image represents upright phonation. Three runs are shown and measures are marked in different colors:

1. Diaphragm anterior (DPH_ant_ ) = blue & posterior DPH_post_ = green
2. anterior-posterior lung diameter at the height of the 3th rib (apD_3R_) = pink & 5^th^ rip (apD_5R_) = lilac
3. Highest point/medial part of diaphragm (DPH_med_) = red & anterior-posterior diameter from DPH cupola to the back (apD_DPH_) = yellow

Additionally, the corresponding movement curves including maximum inspiration and expiration are presented in the right part of the video. At the end of each run, an overlay image is displayed showing the lung configuration at the beginning and end of phonation, as well as upright and supine phonation.

**Supplementary Figure S12**

**No major difference in curve progression between 5 different tasks for upright rMRI and supine hMRI**, Detailed descriptions of the tasks can be found in supplementary table S2. Upright rMRI data is displayed on the left, supine hMRI data on the right. Mean normalized curve amplitude (A_norm_) and standard error is displayed on the y-axis and normalized time (t_norm_) is displayed on the x-axis. Different locations are displayed in different colours.
