# Supplementary figures and images for: The influence of gravity on respiratory kinematics during phonation measured by dynamic magnetic resonance imaging
Source: Sci Rep. 2021 Nov 25;11:22965. doi: 10.1038/s41598-021-02152-y (PMC8617256; doi:10.1038/s41598-021-02152-y)

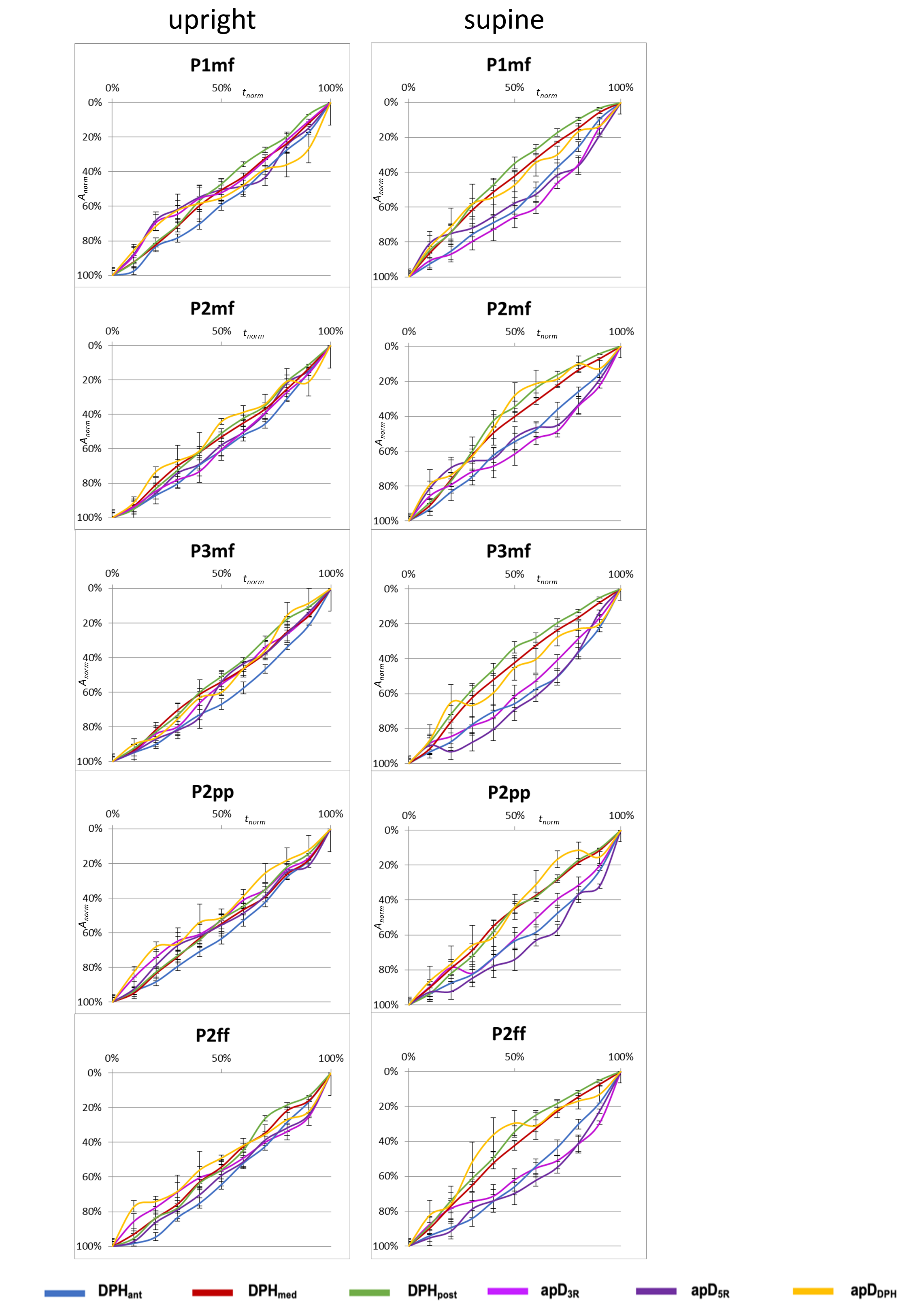

Supplement: Supplementary file 3 — Supplementary Figure 1. [file 41598_2021_2152_MOESM3_ESM.tif]
